# A miRNA-Based Prognostic Model to Trace Thyroid Cancer Recurrence

**DOI:** 10.3390/cancers14174128

**Published:** 2022-08-26

**Authors:** Eman A. Toraih, Manal S. Fawzy, Bo Ning, Mourad Zerfaoui, Youssef Errami, Emmanuelle M. Ruiz, Mohammad H. Hussein, Muhib Haidari, Melyssa Bratton, Giovane G. Tortelote, Sylvia Hilliard, Naris Nilubol, Jonathon O. Russell, Mohamed A. Shama, Samir S. El-Dahr, Krzysztof Moroz, Tony Hu, Emad Kandil

**Affiliations:** 1 Division of Endocrine and Oncologic Surgery, Department of Surgery, School of Medicine, Tulane University, New Orleans, LA 70112, USA; 2 Genetics Unit, Department of Histology and Cell Biology, Faculty of Medicine, Suez Canal University, Ismailia 41522, Egypt; 3 Department of Medical Biochemistry and Molecular Biology, Faculty of Medicine, Suez Canal University, Ismailia 41522, Egypt; 4 Department of Biochemistry, Faculty of Medicine, Northern Border University, Arar P.O. Box 1321, Saudi Arabia; 5 Department of Biochemistry and Molecular Biology, School of Medicine, Tulane University, New Orleans, LA 70112, USA; 6 Department of Pathobiological Sciences, School of Veterinary Medicine, Louisiana State University, Baton Rouge, LA 70803, USA; 7 School of Medicine, Tulane University, New Orleans, LA 70112, USA; 8 Biospecimen Core Laboratory, Louisiana Cancer Research Center, New Orleans, LA 70112, USA; 9 Section of Pediatric Nephrology, Department of Pediatrics, School of Medicine, Tulane University, New Orleans, LA 70112, USA; 10 Endocrine Oncology Branch, National Cancer Institute, National Institute of Health, Bethesda, MD 20814, USA; 11 Division of Head and Neck Endocrine Surgery, Department of Otolaryngology-Head and Neck Surgery, Johns Hopkins, Baltimore, MD 21287, USA; 12 Department of Pathology and Laboratory Medicine, Tulane University School of Medicine, New Orleans, LA 70112, USA

**Keywords:** recurrence, progression, microRNA, thyroid cancer

## Abstract

**Simple Summary:**

Some thyroid tumors elected for surveillance remain indolent, while others progress. The mechanism responsible for this difference is poorly understood, making it challenging to devise patient surveillance plans. Early prediction is important for tailoring treatment and follow-up in high-risk patients. The aim of our study was to identify predictive markers for progression. We leveraged a highly sensitive test that accurately predicts which thyroid nodules are more likely to develop lymph node metastasis, thereby improving care and outcomes for cancer patients.

**Abstract:**

Papillary thyroid carcinomas (PTCs) account for most endocrine tumors; however, screening and diagnosing the recurrence of PTC remains a clinical challenge. Using microRNA sequencing (miR-seq) to explore miRNA expression profiles in PTC tissues and adjacent normal tissues, we aimed to determine which miRNAs may be associated with PTC recurrence and metastasis. Public databases such as TCGA and GEO were utilized for data sourcing and external validation, respectively, and miR-seq results were validated using quantitative real-time PCR (qRT-PCR). We found miR-145 to be significantly downregulated in tumor tissues and blood. Deregulation was significantly related to clinicopathological features of PTC patients including tumor size, lymph node metastasis, TNM stage, and recurrence. In silico data analysis showed that miR-145 can negatively regulate multiple genes in the TC signaling pathway and was associated with cell apoptosis, proliferation, stem cell differentiation, angiogenesis, and metastasis. Taken together, the current study suggests that miR-145 may be a biomarker for PTC recurrence. Further mechanistic studies are required to uncover its cellular roles in this regard.

## 1. Introduction

Among cancers, thyroid carcinoma (TC) is the most prevalent endocrine tumor with the largest increasing annual incidence rate in the United States (U.S.). TC is projected to surpass colorectal carcinoma as the fourth most common cancer overall by 2030 [1]. Owing to its low mortality rate and prolonged follow-up management, TC imposes a high healthcare burden and financial cost on the expanded survivor pool [2,3,4]. Papillary thyroid carcinomas (PTC) are the most common histological subtype, comprising 85% of all differentiated thyroid cancers. Although PTCs are the least aggressive thyroid cancers and have an 80–95% five-year overall survival rate, these tumors can metastasize into lymph nodes and various organs, leading to poorer prognosis. Up to 30% of patients experience recurrence after initial treatment, leading to an over 60% increase in mortality rate [5,6,7,8,9]; thus, a key issue in managing patients with PTC is minimizing the morbidity and mortality associated with disease recurrence.

Clinicopathological risk stratification by the American Thyroid Association (ATA) has been used to classify well-differentiated thyroid cancer (DTC) cohorts to guide the management of patients [10,11]. However, it is a one-time assessment at the time of initial diagnosis and relies on genomic mutation and the final pathological results of tissue biopsies. Like other cancers, repeated tissue biopsy is not feasible following operative excision. The current follow-up modalities, including basal and recombinant human thyrotropin (rhTSH)-stimulated serum thyroglobulin measurements, as well as neck ultrasonography, offer only a snapshot of the disease at a single point in time, have limited sensitivity and specificity, and are poor predictors of progression [12]. Radioiodine whole-body scintigraphy (WBS), which takes advantage of the high avidity of radioiodine in the functioning thyroid tissues, has been used for the detection of DTC; however, radioiodine uptake is not specific for thyroid tissue, with the potential of false-positive results [13]. Additionally, post-ablative non-stimulated Tg have been proposed as a valuable prognostic marker during follow-up [14], with postoperative decline showed good prognostic efficacy for a tumor-free status [15]. Nevertheless, undetectable serum thyroglobulin levels are found in more than 40% of DTC patients with residual or structural recurrence [16,17,18]. Thyroglobulin has been reported as a predictor for treatment efficacy during ablative radioiodine treatment; however, some patients still exhibit elevated levels even after receiving adjuvant radioactive iodine therapy. Moreover, thyroglobulin autoantibodies, found in ~25% of patients and known to interfere with the measurement of thyroglobulin, render tests unreliable [19,20]. Conversely, unnecessary treatment by radioactive iodine or high doses of thyroid hormone replacement can elicit secondary malignancies [21,22,23,24,25,26,27,28,29], adverse cardiovascular or skeletal outcomes [30,31,32], and mortality [31]. Risk stratification to decide which patients should be treated with adjuvant radioiodine therapy or extensive postoperative surveillance following surgery remains highly challenging, as it is difficult to accurately predict which tumors will remain indolent. Hence, there is an urgent need to establish a well-validated ancillary molecular predictive marker that accurately identifies patients at risk of progression. In the absence of such an assay, prognostication will continue to be a challenging practice that puts clinicians at risk of overtreating low-risk patients or undertreating high-risk patients.

Despite decades of research, a simple and universal test to identify cancer recurrence at its earliest stages remains elusive. The accurate and timely prognostication of cancer is a prerequisite for efficient postoperative surveillance and is critical for improving disease-free and overall survival [33]. The ideal test would eliminate the need for biopsies and other invasive and risky procedures to expose cancers in the most inaccessible parts of the body. Recent advances have suggested that liquid biopsies have the potential to detect most cancers at early stages when treatment is more likely to be effective [34]. Liquid biopsies have transformed clinical practice by detecting minimal residual disease after surgery and complementing imaging to monitor progression and treatment response [35,36]. The procedure is non-invasive, easily repeatable, detects tumor evolution and heterogeneity, and can predict clinical outcomes [35].

Epigenetics is inextricably linked to the malignant phenotype [37]. Several molecular studies have investigated the roles of various non-coding RNAs along with genetic and epigenetic modifications in cancer recurrence and progression [37]. Cancer reprogramming confers drastic changes in chromatin structure and transcriptomic profiles, which instruct both cell fate and state transition. Cancer-related epigenomic abnormalities arise in advance of morphological alterations [35]; therefore, TC cells with invasive potential exhibit modifications in their general gene expression pattern before morphological alterations of the lesion are even visible. Testing molecular biomarkers before changes are clinically or radiologically evident would facilitate the early detection of minimal disease or recurrence.

Small non-coding microRNAs (miRNAs) are essential for post-transcriptional regulation of gene expression and are attracting increasing attention because of their association with tumor progression. Enrichment and depletion of miRNAs lead to deregulated co-expression of miRNA target genes and disrupted cellular biological processes in cancer [38,39]. They can be secreted into circulation and exist in remarkably stable forms under extreme conditions such as RNase exposure, multiple freeze–thaw cycles, and extreme pH [34,40]. Like intercellular miRNAs, circulating miRNAs participate in the regulation of numerous biological processes and are expressed aberrantly under pathological status. Deregulation of circulating miRNAs is associated with the initiation and progression of cancer; therefore, high-efficacy, low-cost detection of miRNAs may aid in non-invasive diagnostics and prognostics.

The aim of this paper is to create a miRNA-based model to track long-term prognosis and recurrence of PTC. We used microRNA sequencing analysis in TCGA and GEO datasets to explore miRNA expression profiles of PTC patients. The results were validated using quantitative real-time PCR (qRT-PCR). We found miR-145 to be significantly downregulated in tumor tissues and blood. Deregulation was significantly related to clinicopathological features of PTC patients including tumor size, lymph node metastasis, TNM stage, and recurrence. Both in vivo and in vitro studies were reviewed to determine the putative role of miR-145 in tumor recurrence, and our results suggest that miR-145 may be a biomarker for PTC recurrence.

## 2. Methods

### 2.1. Identification of Recurrence-Specific Regulatory Network from TCGA Database

#### 2.1.1. Data Source and Pre-Processing

Transcriptomic sequence data from 495 thyroid cancer patients were retrieved from the Genomic Data Commons (GDC) data portal for The Cancer Genome Atlas thyroid cancer dataset (TCGA-THCA) (https://www.cancer.gov/about-nci/organization/ccg/research/structural-genomics/tcga, accessed on 1 November 2020). A total of 1035 microRNAs from miRNA-seq and 20,531 lncRNAs and mRNAs from RNA-seq were included. Clinical and pathological data, molecular landscape, and survival information were obtained from cBioPortal for Cancer Genomics (https://www.cbioportal.org, accessed on 10 February 2020) and FireBrowse (http://www.firebrowse.org/, accessed on 10 February 2020). Outcomes of interest included disease recurrence and/or progression. Patients with incomplete recurrence data or unmatched microRNA and RNA sequencing data were excluded, leaving 467 matched cases for analysis. TCGA-THCA cohorts were classified according to 2015 ATA risk stratification for structural disease recurrence into low, intermediate, or high-risk groups. Demographic, oncologic, and clinicopathologic data were compared between recurrent and non-recurrent groups.

#### 2.1.2. Identification of Differentially Expressed Genes (DEG) and miRNAs (DEmiR) in Recurrence

The gene read counts were first filtered for low abundance and low variance transcripts. Mapped reads were summarized into a gene level count using the median estimates. EdgeR was applied to identify the differentially expressed microRNAs (DEmiRs) and RNA genes (DERs) in recurrent compared with non-recurrent groups. Raw data were normalized using log2-counts per million. The significance threshold was set at a false discovery rate (FDR, the q-value) < 0.05 and a |log2 fold change (FC)| > 1.0. Box plots for global transcriptomic signature and volcano plots for genes were generated. Spearman’s correlation analysis was performed to examine co-expression between significant DEGs and DEmiRs, and a correlation matrix was generated.

#### 2.1.3. Functional Enrichment Analysis

The Database for Annotation, Visualization, and Integrated Discovery (DAVID) online tool v6.8 (https://david.ncifcrf.gov/, accessed on 1 July 2021) and DIANA-mirPath v3.0 (https://dianalab.e-ce.uth.gr/html/mirpathv3/index.php?r=mirpath, accessed on 1 July 2021) were utilized for Gene Ontology (GO) analysis to annotate genes and to define the functions of DEG into three domains: cellular component, biological process, and molecular function. Subsequently, KEGG enrichment analysis was conducted to discover the potential signaling pathways in which DEGs were involved. The cut-off value of significant GO terms and KEGG pathway was *p* < 0.05.

#### 2.1.4. Predictive Performance of DEG and DEmiR

Association analysis was performed for the 8 miRNAs and 16 genes using Mann–Whitney U test in the TCGA cohort. Receiver operating characteristics (ROC) curve was employed to estimate their prognostic performance with various clinical, genomic mutation, and pathological data. The optimum threshold level was selected using the Youden index. Area under the curve (AUC), test accuracy, sensitivity, specificity, and diagnostic odds ratio measures were calculated. DEmiRs/DEGs with AUC > 0.75 and *p* < 0.05 were selected for further downstream analysis. Univariate and multivariate Cox proportionate hazards regression analyses were employed to predict disease recurrence. Hazard ratios and 95% confidence intervals were reported. *p*-values were corrected for multiple-hypothesis testing using the Benjamini–Hochberg correction, with a significance threshold of FDR < 0.05.

### 2.2. External Validation in GEO

#### 2.2.1. Expression Pattern of miR-145 in Tissues of Cancer Patients

Analysis of 145 microarray and miRNA-sequencing datasets from public repositories including Gene Expression Omnibus (GEO), Sequence Read Archive (SRA), ArrayExpress, and The Cancer Genome Atlas (TCGA) stored in the database of Differentially Expressed MiRNAs in human Cancers (dbDEMC) v3.0 webtool (https://www.biosino.org/dbDEMC/index, accessed on 1 August 2021) depicted the expression level of miR-145-5p in (1) various types of tumor tissues compared with controls and (2) aggressive versus non-aggressive samples such as metastasis versus none, high versus low grade, or poor versus good outcome.

#### 2.2.2. Expression Pattern of miR-145 in Liquid Biopsies of Cancer Patients

Circulatory and exosomal miRNA-sequencing analysis of 14 GEO datasets (E_MTAB_1454, GSE106817, GSE112264, GSE112840, GSE113486, GSE113740, GSE122497, GSE139031, GSE31568, GSE39845, GSE59856, GSE65071, SRP078325, and SRP262521) in the circulation of cancer patients.

### 2.3. External Validation in Independent Cohorts

#### 2.3.1. Ethical Statement

Ethical approval was provided by the Institutional Review Board, Tulane University School of Medicine, United States (#2021–1214) and the Medical Research Ethics Committee, Suez Canal University School of Medicine, Egypt (#4344). The study followed “Declaration of Helsinki” guidelines.

#### 2.3.2. Human Specimens

A total of 484 thyroid specimens (242 cancer tissues and their paired non-cancer adjacent tissues) were analyzed. These included 64 paired fresh frozen samples from the Louisiana Cancer Research Center (LCRC), New Orleans, Louisiana, United States, and 178 paired formalin-fixed paraffin-embedded (FFPE) archival samples from three Pathology Labs of (i) El-Bayan, Portsaid City; (ii) Mansoura University, Mansoura City; and (iii) Suez Canal University, Ismailia City, Egypt. In addition, 111 blood samples (64 thyroid cancer, 36 benign non-toxic multinodular disease, and 11 control subjects) were recruited from Tulane Medical Center, New Orleans, LA, United States. Tissue samples were obtained during surgical resection, and fasting blood samples were collected before thyroidectomy. Deidentified samples were processed and linked to their demographic, pathological, and clinical information.

The current study investigated patients diagnosed with well-differentiated thyroid cancer (papillary TC or follicular TC) according to the International Classification of Oncological Diseases, 4th edition, who underwent thyroidectomy and/or lobectomy and did not receive any treatment before operative resection. There was no restriction for age, sex, race, ethnicity, national origin, language, religion, pregnancy, socioeconomic status, or disability. The exclusion criteria included (a) poorly differentiated thyroid carcinoma, anaplastic (undifferentiated) carcinoma, medullary thyroid cancer, Hürthle cell thyroid carcinoma, thyroid lymphoma, thyroid cancer arising from a thyroglossal duct cyst, thyroid cancer in malignant struma ovarii, or secondary carcinomas; (b) patients with a history of primary cancer in other organs; (c) patients with incomplete follow-up or missing clinical data; (d) cohorts with unmatched paired tissue samples; or (e) no available archived paraffin blocks and/or blocks with insufficient tissue to perform immunohistochemical studies.

#### 2.3.3. Clinical Assessment and Outcomes

Baseline demographic and clinicopathological characteristics were obtained from electronic medical records. Demographic data included age at diagnosis, sex, race, ethnicity, and year of diagnosis. For risk assessment, body mass index (BMI), smoking and alcohol intake, history of radiation exposure, occupational hazards, and family history of cancer or previous thyroid diseases were evaluated. Pathological characteristics included tumor side and laterality, histopathological subtype (PTC or FTC) and variant, TNM (tumor, node, and metastasis) stage, and extrathyroidal extension (ETE). Sonographic features and genomic mutation reports were collected if available. Patients with concomitant Hashimoto’s thyroiditis were identified. Laboratory results for differential white blood cell count, hepatitis C virus, thyroglobulin, and autoantibodies were collected. The type of surgical resection (unilateral, subtotal, or total thyroidectomy), extent of neck dissection (central, unilateral, or bilateral block neck dissection), thyroxine intake, and use of radioactive iodine or other adjuvant treatment modalities (e.g., external beam radiotherapy) were reported. Additional lines of treatment received during the postoperative surveillance period were obtained.

**Figure 1:**
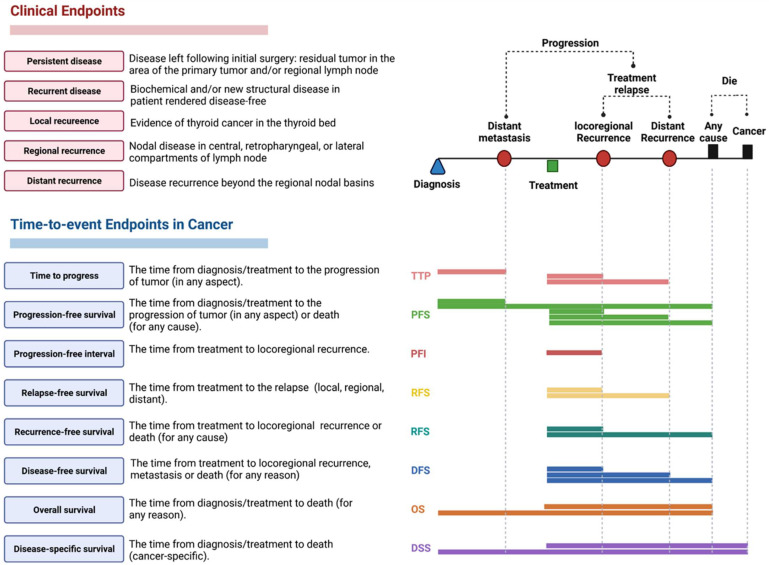
Time-to-event endpoint metrics for survival analysis; definition of the assessment of time-to-event endpoints and clinical outcomes in cancer.

Post-thyroidectomy, patients were monitored every six months. They underwent clinical examination, imaging studies, and serum thyroglobulin level estimation. Relapse, recurrence, progression, and death were reported at follow-up (Figure 1). Patients fulfilled the 2015 ATA intermediate-risk criteria: exhibiting aggressive histology (tall cell, solid, hobnail), microscopic extrathyroidal extension (peri-thyroid fat, strap muscles), vascular invasion, or >5 involved lymph nodes (0.2–3 cm). Our primary outcomes were the presence of persistent or recurrent disease. Persistent disease was identified as residual tumor in the area of the primary tumor or regional nodes. Clinical recurrence was defined as the reappearance of malignancy in the thyroid bed (local recurrence), lymph node (regional recurrence), or distant metastasis (distant recurrence) following initial surgery. No clinical evidence of disease (NCED) was defined as the absence of disease at the end of the follow-up period, based on physical examination and imaging studies. Progression-free survival (PFS) was defined as the time from initial diagnosis to the progression of tumor (distant metastasis, local recurrence, and regional or distant recurrence) or death by any cause. Disease-free survival measured the period from initial surgery to the time of recurrence (local, regional, or distant) or death by any cause, while overall survival referred to the time from initial surgery to death by any cause, regardless of the presence or absence of evidence of recurrence/progression. Outcomes were assessed as binary and time-to-event variables.

#### 2.3.4. Histopathological Assessment

FFPE blocks of tissue specimens were reviewed by a pathologist to distinguish between cancer and non-cancer regions and assess the percentage of tumor cells and normal cells. The following microscopic parameters were assessed via hematoxylin and eosin (H & E) staining: histopathological variant, TNM tumor stage according to the American Joint Committee on Cancer (AJCC, 8th edition), focality, ETE, lymphatic invasions, extranodal extension, perineural invasion, and safety margin. FFPE sections were cut of 4-micron in thickness in separate Eppendorf tubes (cancer and non-cancer) for molecular analysis. Fresh frozen tissues were cryosectioned and stained with the Histogene™ laser capture microdissection (LCM) frozen section staining kit and micro-dissected using the Arcturus LCM instrument. Only tissues containing <20% necrosis were subjected to nucleic acid extraction.

#### 2.3.5. RNA Extraction and Quantification of miR-145-5p in Tissues and Liquid Biopsies

Following xylene deparaffinization, total RNA (including small RNAs) was isolated from 178 paired cancer and non-cancer FFPE tissues using Qiagen miRNeasy FFPE Isolation kit (catalog number 73504, Qiagen, Germantown, MD, USA). Total RNA including miRNA was isolated from the 64 paired frozen samples using Arcturus^®^ PicoPure^®^ Frozen RNA Isolation Kit (catalog number KIT0204, ThermoFisher, Waltham, MA, USA), according to the manufacturer’s protocol. Circulatory miRNA was isolated from 183 archived peripheral plasma samples using total exosome RNA and protein isolation kit (catalog number 4478545, Thermo Fisher Scientific, Waltham, MA, USA) and Qiagen miRNeasy Serum/Plasma Kit (catalog no. 217184, Qiagen). The total RNA quality and quantity were assessed via absorbance spectrophotometry on a Nanodrop-1000 spectrophotometer at the wavelength-dependent extinction coefficient of 33 (Thermo Scientific, Wilmington, DE, USA) and Qubit™ fluorometer (Invitrogen, Waltham, MA, USA).

RNA (10 ng) was converted to complementary DNA (cDNA) using TaqMan MiRNA Reverse Transcription (RT) kit (P/N 4366596, Applied Biosystems, Foster City, CA, USA); Thermo Fisher, CA, USA), and 5× specific stem-loop primers or endogenous control primers (RNU6B, catalog number 001039) for normalization were used separately. Reverse transcription (RT) was carried out in a T-Professional Basic, Biometra PCR system (Biometra, Goettingen, Germany) at the following amplification conditions: 16 °C for 30 min, 42 °C for 30 min, and 85 °C for 5 min, and then held at 4 °C. For quality control assessment, miR-23a, miR-30c, miR-103, miR-191, and miR-451 were tested for hemolysis, and the RNA spike-ins UniSp101, UniSp100, and UniSp6 were assayed to screen the inhibition of enzymatic reactions.

Real-time quantitative polymerase chain reaction (PCR) was followed using Universal Master Mix (catalog number 4440042) and TaqMan assay for miR-145-5p (Assay ID: 002278, Applied Biosystems, Thermo Fisher Scientific Inc.) in StepOne™ Real-Time PCR System (Applied Biosystems) and QuantStudio 6 Flex (Applied, Foster City, CA, USA). Reactions were run in triplicate under the following conditions: 95 °C for 10 min, followed by 45 cycles of 92 °C for 15 s and 60 °C for 1 min. Appropriate negative controls were included in each run (no template and no enzyme samples), and duplicate PCR runs were performed. The Minimum Information for Publication of Quantitative Real-Time PCR Experiments (MIQE) guidelines were followed during the experiments. Fold changes were estimated via the Livak method based on the quantification (threshold) cycle (Cq or CT) value: relative gene expression = 2^−ΔΔCq^. Standard deviation >2.0 was set as an outlier.

### 2.4. Systematic Review

A systemic review on miR-145 in thyroid cancer was performed on 26 June 2022 using PubMed, Embase, Google Scholar, and genomic public repositories following PRISMA guidelines. The keywords “(miR-145 OR microRNA-145 OR MIR145) AND (thyroid)” were used in the search process. All human, in vitro, and in vivo studies were included, without time or language restriction. Manual searches of relevant bibliographic articles were performed. Data abstraction was performed in Microsoft Excel (Muhib MH and ET) and stratified by the functional role of a biomarker.

### 2.5. Functional Role of miR-145

Cancer Hallmarks Analytic tools (https://chat.lionproject.net) were used to define miR-145-related hallmarks based on online publications. Gene targets of miR-145-5p were identified using miRTargetLink 2.0 (https://ccb-compute.cs.uni-saarland.de/mirtargetlink2). Starbase software (http://starbase.sysu.edu.cn/mirLncRNA.php) was used to identify competing endogenous RNA regulatory network sponging miR-145. Diana miRPath v3.0 (https://dianalab.e-ce.uth.gr/html/mirpathv3/) and ingenuity pathway analysis software (https://reports.ingenuity.com/) were used to investigate enriched KEGG pathways and Gene Ontology terms. Harmonizome database (https://maayanlab.cloud/Harmonizome/) was used to identify CHEA transcription factor binding sites and targets of the promoter of MIR145 gene in low- and high-throughput functional studies. All previous online tools were last accessed on 17 April 2022)

### 2.6. Statistical Analysis

Statistical analysis was conducted using the R programing language (v4.1.2), Scikit-learn v0.22 package in Python (Python Software Foundation, Wilmington, DE, USA), GraphPad Prism v7.0 (San Diego, CA, USA), SPSS (IBM, V27. New York, NY, USA: IBM Corp), and STATA v16 (StataCorp. College Station, TX, USA), in addition to OmicSoft and IPA (Qiagen, Hilden, Germany). The estimated power is 90% for a minimum of 228 TC patients to predict disease progression, medium effect size = 0.5, and alpha error probability = 0.05, using G*Power version 3.1.9.2 (http://www.gpower.hhu.de/) (accessed on 10 February 2022). For continuous variables, means and standard deviations or medians with interquartile ranges were calculated. For categorical variables, absolute numbers with percentages were recorded. Categorical variables were compared using chi-square (χ^2^) or Fisher’s exact tests where appropriate, while the Student’s *t*-test, Wilcoxon matched-pairs signed rank test, or Mann–Whitney U test were used to compare continuous variables. Changes in miRNA expression were calculated via the LIVAC method based on the Cq value; 2^−ΔΔCq^ formula. Wilcoxon matched-pairs signed-rank test was used for comparison between cases and controls. Correlations between blood and tissue results were compared using Spearman’s correlation test. Diagnostic statistics such as specificity, sensitivity, positive and negative predictive values, ROC analysis with AUC estimation, DeLong test, and F-test were performed using pROC R package to determine the performance of the miRNA in blood and tissues. Multivariate regression analysis was used to build a predictive model for recurrence/progression in TC patients at the time of surgery using selected clinical, pathological, and molecular data. Biological variability by age, gender, and race was adjusted. The prognostic risk scores for each patient were calculated based on a linear combination of the miRNA expression level and demographics weighted by the regression coefficient derived from the regression. Prediction accuracy was assessed using Harrell’s concordance index (C-index), Brier score (BS), and Hosmer–Lemeshow test using DescTools R package and Bootstrap 1000. Discrimination and calibration were evaluated. Decision curve analyses (DCAs) were performed to test the clinical utility of the Cox nomogram model. Principal component analysis and hierarchical clustering were employed for risk stratification. Disease-free and overall survival analysis was performed, and Kaplan–Meier curves were plotted. Log rank (Mantel–Cox) test was used. The Survminer package (https://cran.r-project.org/web/packages/survminer/index.html, accessed on 17 April 2022) for R was applied to determine the optimal cut-off value to divide patients into two groups, high or low gene expression, based on receiver operating characteristic. A two-tailed *p*-value of < 0.05 was considered significant.

## 3. Results

### 3.1. Network Discovery from TCGA Cohorts

#### 3.1.1. Characteristics of TCGA Cohorts

A total of 467 cancer patients (104 FTC and 363 PTC) were included in the analysis. Mean age was 46.0 years (34.0–57.0) and 73.2% were female. Of all patients, 27.2% were classified as low risk, 49.9% were classified as intermediate risk, and 22.9% were classified as high risk. Characteristics of recurrent (*n* = 44) and non-recurrent (*n* = 423) groups are shown in Table 1. Patients in the recurrent group were more likely to be older (*p* = 0.040) and white (*p* = 0.018). A higher prevalence of recurrence was found in patients with tumor stage T3/T4 (*p* = 0.009), distant metastasis (*p* < 0.001), and TERT mutation (*p* = 0.011). The median overall survival was 31.0 months (IQR = 17.4–51.9), with longer follow-up of the recurrent group (*p* = 0.006).

#### 3.1.2. Expression Signature for Recurrence

**Figure 2:**
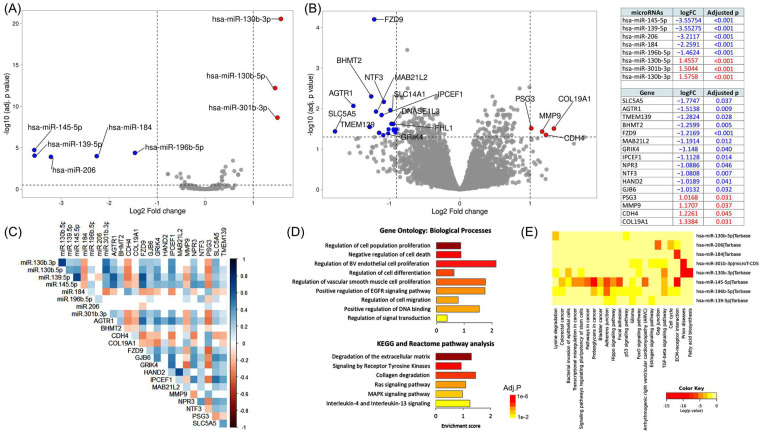
Identification of recurrence-specific differentially expressed genes. RNA-seq and microRNA-seq analysis of 467 tissue specimens of PTC patients in The Cancer Genome Atlas (TCGA) database integrated with clinical data, pathological data, molecular landscape, and survival information. By comparing the transcriptome signature of 46 recurrent and 441 disease-free patients, we identified a panel of eight deregulated microRNAs and 16 deregulated mRNAs associated with disease recurrence and/or progression. (**A**) Volcano plot and table showing differentially expressed microRNAs in recurrent cohorts compared with the non-recurrence group. The significance threshold was set at a false discovery rate (FDR) < 0.05 and a |log2 fold change (logFC)| > 1.0. Three upregulated genes in red and five downregulated genes in blue are shown. (**B**) Volcano plot and table showing differentially expressed mRNAs/lncRNAs in recurrent cohorts compared with the non-recurrence group. Four upregulated genes in red and 12 downregulated genes in blue are shown. (**C**) Co-expression analysis of genes and miRNAs. Spearman’s correlation analysis was performed. In the correlation matrix, red color shows a positive correlation and blue shows a negative correlation. (**D**) Functional enrichment analysis of DEGs. (**E**) Pathway enrichment analysis of DEmiRs using Diana lab tools. Abbreviations: solute carrier family 5 member 5 (*SLC5A5*), angiotensin II receptor type 1 (*AGTR1*), transmembrane protein 139 (*TMEM139*), betaine--homocysteine S-methyltransferase 2 (*BHMT2*), frizzled class receptor 9 (*FZD9*), mab-21 like 2 (*MAB21L2*), glutamate ionotropic receptor kainate type subunit 4 (*GRIK4*), interaction protein for cytohesin exchange factors 1 (*IPCEF1*), natriuretic peptide receptor 3 (*NPR3*), neurotrophin 3 (*NTF3*), heart and neural crest derivatives expressed 2 (*HAND2*), gap junction protein beta 6 (*GJB6*), pregnancy-specific beta-1-glycoprotein 3 (*PSG3*), matrix metallopeptidase 9 (*MMP9*), cadherin 4 (*CDH4*), and collagen type XIX alpha 1 chain (*COL19A1*).

Based on microRNA-seq analysis, we identified five downregulated and three upregulated differential miRNAs in the recurrent group (Figure 2A). Specifically, hsa-miR-145-5p was the most downregulated microRNA in recurrent patients (FC = −3.55, *q* = 1.86 × 10^−5^), followed by hsa-miR-139-5p (FC = −3.55, *q* = 8.93 × 10^−5^), hsa-miR-206 (FC = −3.21, *q* = 1.26 × 10^−4^), hsa-miR-184 (FC = −2.25, *q* = 1.03 × 10^−4^), and hsa-miR-196b-5p (FC = −1.46, *q* = 4.18 × 10^−5^). In contrast, hsa-miR-130b-3p (FC = 1.57, *q* = 2.76 × 10^−21^), hsa-miR-301b-3p (FC = 1.50, *q* = 2.31 × 10^−9^), and hsa-miR-130b-5p (FC = 1.45, *q* = 6.16 × 10^−13^) were upregulated.

Analysis of RNA-seq data revealed 12 downregulated and four upregulated protein-coding genes in the recurrence group (Figure 2B). Among the negatively regulated genes are sodium/iodide cotransporter (SLC5A5), which mediates iodide uptake in the thyroid gland (FC = −1.77, *q* = 0.036); angiotensin II receptor type-1 (AGTR1), which regulates blood pressure and aldosterone secretion (FC = −1.51, *q* = 0.008); transmembrane protein 139 gene, involved in cellular trafficking of proteins (TMEM139) (FC = −1.28, *q* = 0.028); and betaine--homocysteine S-methyltransferase 2 (BHMT2) (FC = −1.25, *q* = 0.005), involved in the regulation of homocysteine metabolism via converting homocysteine to methionine using S-methyl methionine (SMM) as a methyl donor. In contrast, collagen alpha-1 (XIX) chain (COL19A1), one of the fibril-associated collagens that serve to maintain the integrity of the extracellular matrix (EMC), was the most upregulated gene (FC = 1.33, *q* = 0.031). Another protein necessary for cell adhesion, cadherin-4 (CDH4), was significantly overexpressed in recurrence cases (FC = 1.22, *q* = 0.044). Of the upregulated genes, matrix metalloproteinase-9 (MMP9) is a zinc-dependent endopeptidase and the major protease in EMC degradation and leukocyte migration (FC = 1.17, *q* = 0.036). Pregnancy-specific beta-1-glycoprotein 3 (PSG3), one of the immunoglobulin superfamily of genes shown to function as an adhesion recognition signal for several integrins, was slightly upregulated (FC = 1.01, *q* = 0.030). The co-expression matrix in Figure 2C shows weak correlations between genes and miRNAs.

#### 3.1.3. Functional Enrichment Analysis

As depicted in Figure 2D, the top biological processes included (1) regulation of cell population proliferation (GO:0042127; *q* = 1.04 × 10^−6^) through NGFR, TIMP1, NPR3, NTRK2, MAB21L2, FZD9, GJB6, HAND2, NTRK3, AGT, MMP9, NTF3, and AGTR1 genes; (2) negative regulation of cell death (GO:0060548; *q* = 8.2 × 10^−4^) via eight genes (NGFR, TIMP1, NTRK2, FZD9, HAND2, NTRK3, MMP9, and NTF3); (3) regulation of cell differentiation (GO:0045595; *q* = 0.0036) by enrichment of NGFR, NTRK2, HAND2, NTRK3, AGT, NTF3, AGTR1, and CDH4; and (4) regulation of blood vessel endothelial cell proliferation involved in sprouting angiogenesis (GO:1903587; *q* = 0.0025) via NGFR and AGTR1. KEGG and Reactome pathway analysis showed high enrichment of Ras and MAPK signaling pathway (KEGG: hsa04014 and hsa04010), as well as other cancer-related pathways such as degradation of the extracellular matrix (HSA-1474228; FDR = 0.005; TIMP1, MMP9, COL19A1), signaling by receptor tyrosine kinases (HSA-9006934; FDR = 0.012; NTRK2, NTRK3, MMP9, NTF3), and collagen degradation (HSA-1442490; FDR = 0.022; MMP9, COL19A1). Functional enrichment analysis of miRNAs (Figure 2E) revealed the involvement of the extracellular matrix receptor interaction, TGF-beta signaling pathway, and adherens junction, and miR-145-5p was enriched in almost all significant pathways.

#### 3.1.4. Association of DEG and DEmiR with Clinicopathological Characteristics and Survival Analysis

**Figure 3:**
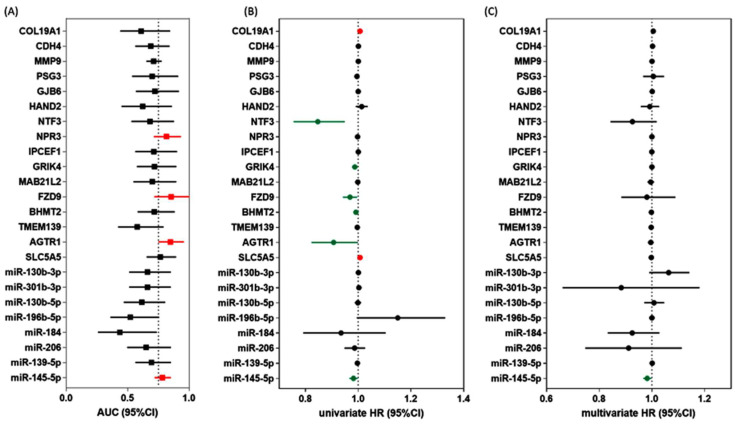
Prognostic and predictive performance of DEG and DEmiR. (**A**) ROC analysis to discriminate patients with recurrence from those with non-recurrence. red indicated higher AUC > 0.75. (**B**) Univariate Cox regression analysis for disease-free survival. Red indicated higher risk, green indicated lower risk of recurrence, while black indicated no significance. (**C**) Multivariate Cox regression analysis for disease-free survival adjusted by age, sex, race, and *BRAF* mutation. Red indicated higher risk, green indicated lower risk of recurrence, while black indicated no significance. Abbreviations: AUC: area under the curve, CI: confidence interval, HR: hazard ratio.

Marker selection for the 8 miRNAs and 16 genes was performed using four statistical tests: Mann–Whitney U test for association analysis, area under the curve (AUC) estimation for receiver operating characteristic analysis to assess prognostic performance, and univariate and multivariate Cox regression analyses to identify the role of each deregulated gene on recurrence. Association analysis with recurrence was significant in 18 markers. ROC analysis showed significance (AUC > 0.75) in 2 miRNAs and 13 genes. The highest AUC was for miR-145-5p, with an AUC of 0.78 (95%CI = 0.71–0.85; 64% sensitivity and 80% specificity), and the best discriminative genes for recurrence were FZD9 (AUC = 0.87, 95%CI = 0.71–0.99), AGTR1 (AUC = 0.85, 95%CI = 0.74–0.95), and NPR3 (AUC = 0.82, 95%CI = 0.71–0.93) (Figure 3A). Univariate regression analyses showed significant results in seven markers (Figure 3B). After adjustment for age, sex, race, and BRAF mutation, multivariate regression analysis revealed an association between miR-145-5p and disease-free survival (Figure 3C).

#### 3.1.5. Low miR-145 Level Is a Poor Prognostic Marker

In TCGA data, low tissue expression of miR-145-5p was associated with more advanced tumor stage T3/4 (*p* = 0.006), distant metastasis (*p* = 0.046), extrathyroidal extension (*p* = 0.026), and *BRAF*^V600E^ mutation (*p* = 0.001). Kaplan–Meier survival curves showed poor survival in patients with low expression (less than median value) (*p* = 0.035).

### 3.2. External Validation in GEO

#### 3.2.1. Expression Pattern of miR-145 in Tumor Tissues of Cancer Patients

**Figure 4:**
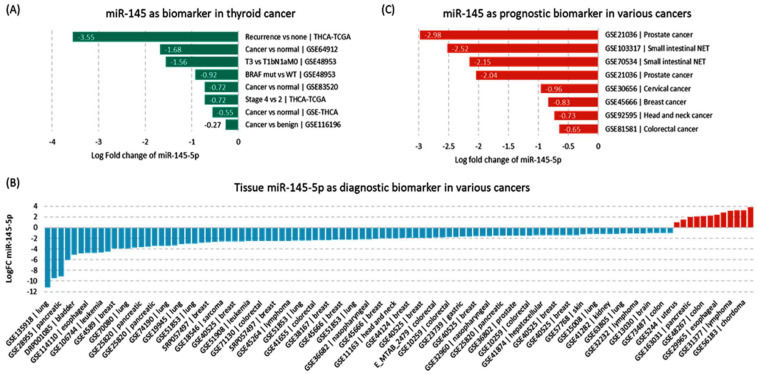
Expression pattern of miR-145-5p in public repositories of cancer. (**A**) Expression in thyroid cancer tissues. (**B**) Expression in other cancer tissues. (**C**) Expression in aggressive versus indolent tissues samples. Data source: dbDEMC database (https://www.biosino.org/dbDEMC/search) (last accessed: 8 August 2022).

Downregulated miR-145-5p was validated in other public datasets for thyroid cancer compared with benign (GSE116196) and adjacent non-cancerous tissues (GSE64912, GSE83520, GSE97070), as well as in aggressive versus non-aggressive tumors (GSE48953) (Figure 4A). In a pan-cancer perspective, 145 datasets were found with available miR-145-5p expression data and 24 with an absolute log fold change below 1.0 were excluded. Fifteen studies were from TCGA, and miR-145 was under-expressed in all of them. The other 106 GEO experiments showed downregulation in 88.6% of the datasets, ranging from −11.21- in lung cancer to −1.03-fold change in colorectal cancer (Figure 4B). Other datasets showed lower expression patterns in aggressive compared with non-aggressive samples (Figure 4C).

#### 3.2.2. Expression Pattern of miR-145 in Liquid Biopsies of Cancer Patients

Through miRNA-sequencing analysis of 14 GEO datasets (E_MTAB_1454, GSE106817, GSE112264, GSE112840, GSE113486, GSE113740, GSE122497, GSE139031, GSE31568, GSE39845, GSE59856, GSE65071, SRP078325, and SRP262521) in the circulation of cancer patients, we observed variability in the direction of miR-145-5p expression pattern even within the same type of cancers (Table 2). Boufraqech et al. identified miR-145 in serum exosome of thyroid cancer patients [41]. Similarly, secreted exosomal-derived miR-145 was upregulated in four head and neck cancer cell lines compared with oral epithelial control cells [42].

### 3.3. Validation in Independent Cohorts

#### 3.3.1. Expression Pattern of miR-145 in Tissues and Blood

**Figure 5:**
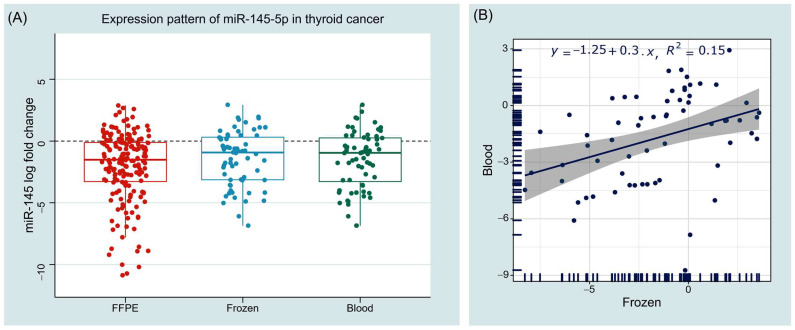
Expression pattern of miR-145 in human tissue and blood samples. (**A**) Boxplots show the log of relative expression levels in cancer samples compared with controls. Wilcoxon matched-pairs signed rank and Mann–Whitney U tests were used. (**B**) Correlation analysis between tissue and blood samples. Spearman’s correlation test was utilized. FFPE: formalin fixed paraffin embedded tissue sections; Frozen: fresh frozen tissues.

As depicted in Figure 5, analyzing thyroid cancer tissue specimens showed downregulation of miR-145-5p in cancer tissues compared with their corresponding paired non-cancer tissues, with median and interquartile values of −1.52 (−3.31–−0.10) in FFPE samples (*n* = 178) and −1.16 (−3.31–0.40) in fresh frozen samples (*n* = 64), *p* < 0.001. Similarly, significantly lower levels were found in plasma of the cancer cohort (*n* = 64) compared with controls (*n* = 47), with a median value of −0.94 (−3.14–0.36), *p* < 0.001. Expression levels in tissues and blood were moderately correlated (*r* = 0.427, *p* < 0.001).

#### 3.3.2. Association with Clinicopathological Characteristics

**Figure 6:**
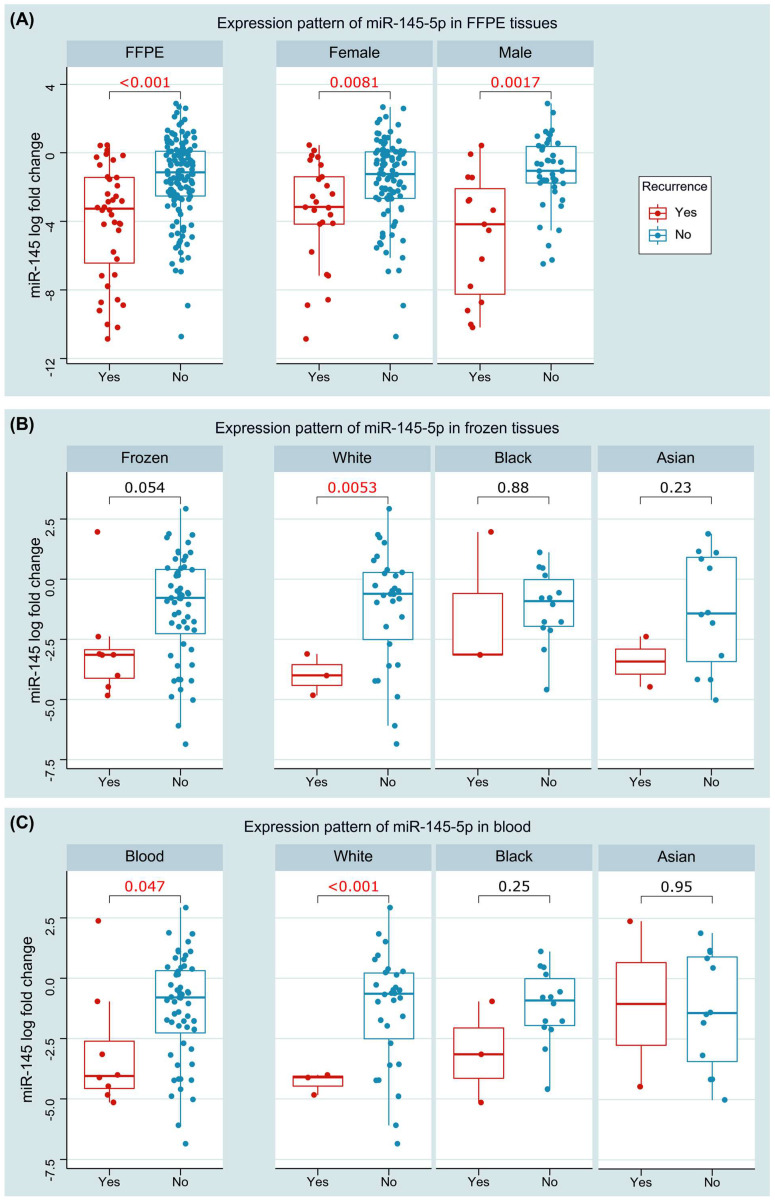
Association of miR145 expression with recurrence. (**A**) Expression in paired FFPE tissues (overall and stratified by sex). (**B**) Expression in paired fresh frozen tissues (overall and stratified by race). (**C**) Expression in blood of cancer and control groups (overall and stratified by race). Wilcoxon matched-pairs signed rank and Mann–Whitney U tests were used. Red values are significant at *p* < 0.05.

Baseline characteristics of the study population are summarized in Table 3. Downregulation of miR-145 was significantly associated with recurrence. Expression levels were lower in tissues of the recurrent cohort (median: −5.2, IQR: −6.39–−1.77) compared with the non-recurrent group (median: −0.90, IQR: −3.16–1.29), *p* < 0.001. Similar findings were found in blood, with lower levels encountered in the recurrence group compared with non-recurrent group (−3.14 (−4.35–−2.17) versus 0.78 (−2.54–0.43), *p* = 0.047). Subgroup analysis showed significant differences between the two groups in both male (*p* = 0.008) and female (*p* = 0.001) patients. Stratification by race showed the association of miR-145 levels with recurrence in the white population (*p* = 0.005 in tissue and <0.001 in blood), sparing Black *(p* = 0.88 and 0.25) and Asian (*p* = 0.25 and 0.95) patients (Figure 6).

Furthermore, tissue miR-145 was associated with clinical stage (*p* = 0.001), larger tumor size (*p* = 0.006), and lymph node metastasis (*p* = 0.008). Circulatory miR-145 was associated with Hashimoto disease (*p* = 0.030) and *TERT* mutation (*p* = 0.004). We did not find an association with other clinical or pathological features.

#### 3.3.3. Predictive Accuracy of miR-145

**Figure 7:**
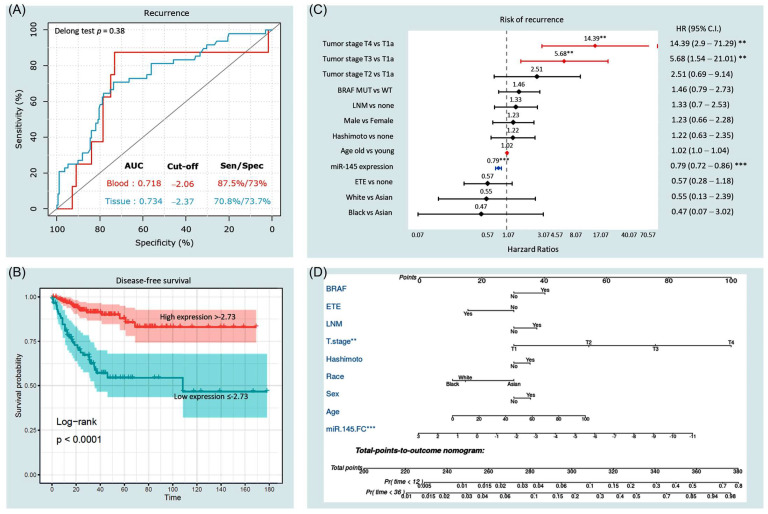
Prognostic value of miR-145 expression. (**A**) Receiver operator characteristic analysis for the role of miR-145-5p in tissues and blood. Area under the curve (AUC) is reported. Sen: sensitivity; spec: specificity. (**B**) Kaplan–Meier survival curve analysis for disease-free survival. Log fold changes in miR-145 in tissues were split into high and low expression groups based on the optimum cutoff value of ROC analysis. Log-rank test was used. (**C**) Predictor risk factors for disease-free survival. Multivariate Cox proportionate hazard regression model was employed. The results are presented as hazard ratio and confidence interval. Red lines have a higher risk of recurrence, while blue lines represent a protective variable. ** indicates *p* < 0.01 and *** indicates *p* < 0.001. (**D**) Prognostic nomogram for predicting recurrence at the time of diagnosis. miR145: microRNA-145-5p expression level as log fold change; ETE: extrathyroidal extension; LNM: lymph node metastasis.

Receiver operator characteristic (ROC) curve analysis demonstrated a good discrimination ability of miR-145 expression level to differentiate between cancer and non-cancer tissues (AUC = 0.734 at cut-off value of −2.37, specificity% = 73.7%, *p* < 0.001) and blood (AUC = 0.718 at cut-off value of −2.06, sensitivity = 87.5%, specificity = 73%, *p* = 0.047) (Figure 7A). Log fold changes in miR-145 in tissues were split into high and low expression groups based on the optimum cutoff value (−2.37) of ROC analysis.

#### 3.3.4. Survival Analysis and Predictors of Progression

The average disease-free survival was 133.4 months (95%CI = 120.9–145.8). Kaplan–Meier survival curves (Figure 7B) demonstrated lower survival probability in patients with low expression levels of miR-145 in cancer tissues (below −2.37) at the time of presentation (mean survival times were 107.2 months ± 10.1 in the low expression group versus 146.0 months ± 5.6 in high expression group, *p* < 0.001). Univariate Cox regression analysis showed that miR-145 (HR = 0.78, 95%CI = 0.72–0.85, *p* < 0.001, concordance = 0.713), age >55 years (HR = 1.02, 95%CI = 1.0–1.03, *p* = 0.032, concordance = 0.60), tumor stage (T3 versus T1: HR = 5.87, 95%CI = 1.78–19.33, *p* = 0.003; T4 versus T1: HR = 9.17, 95%CI = 2.18–38.5, *p =* 0.002, concordance = 0.66), and lymph node metastasis (LNM) (HR = 1.89, 95%CI = 1.05–3.38, *p* = 0.031, concordance = 0.59) were putative risk factors for recurrence. However, after adjustment by multivariate Cox regression analysis (Figure 7C), low miR-145 levels in tissues (HR = 0.79, 95%CI = 0.72–0.86, *p* < 0.001) and blood (HR = 0.74, 95%CI = 95, *p* = 0.048), as well as advanced tumor stage (T4 versus T1: HR = 14.39, 95%CI = 2.9–71.2; T3 versus T1: 5.68, 95%CI = 1.54–21.0), were associated with a risk of mortality. A prognostic nomogram for 1-year and 3-year recurrence-free survival was generated (Figure 7D).

### 3.4. Systemic Review on the Functional Role of miR-145 in Thyroid Cancer

Systematic search; duplicate removal; and screening of titles, abstracts, and full text yielded eight eligible articles [41,43,44,45,46,47,48,49]. The expression of miR-145 was significantly reduced in thyroid cancer compared with normal cells [41]. In contrast, its overexpression in thyroid cancer cell lines resulted in inhibited cell differentiation, proliferation, migration, and invasion. It suppressed VEGF secretion and E-cadherin expression and inhibited the PI3K/Akt pathway by targeting AKT3 [41]. In vivo, miR-145 overexpression decreased tumor growth and metastasis in a xenograft mouse model [41]. Similarly, miR-145 was sponged by the oncogenic lncRNA n384546, which is highly expressed in PTC tissues and cell lines, leading to cancer progression and metastasis. Anti-miR-145 could partially reverse the effects of n384546 knockdown in vitro by regulating AKT3 expression [47]. In another study, miR-145 inhibited the migration and invasion of papillary thyroid carcinoma cells through nuclear factor-κB (NF-κB) pathway regulation [49]. In human tissues, miR-145 was decreased in cancer compared with normal adjacent tissues. Overexpression of miR-145 led to downregulation of its gene target dual-specificity phosphatase 6 (DUSP6) at the protein level and inhibited cell proliferation in TPC1 cells [43]. Furthermore, miR-145 functions as a tumor suppressor in PTC by inhibiting RAB5C [44]. RAB5C is a member of the RAS oncogene family that regulates cell invasion and motility. Zhang et al. found that miR-145 was downregulated in PTCs and negatively correlated with PTC progression and metastasis. MiR-145 inhibited PTC migration and proliferation and promoted apoptosis by directly suppressing RAB5C, suggesting that MiR-145 and RAB5C could serve as therapeutic targets against aggressive PTC cases [44].

Non-coding RNAs (ncRNAs) include long non-coding RNAs (lncRNAs), miRNAs, and circular RNAs (circRNAs). Intracellular circRNAs suppress miRNA by sponging and binding to the miRNA response element (MRE). Overexpressed circNUP214 has been negatively correlated with downregulated miR-145 levels in PTC compared with paratumor tissues [45]. In vitro studies showed that circNUP214 induced tumor cell migration and invasion through miR-145 sponging, releasing the inhibitory effect on zinc finger E-box binding homeobox (ZEB2) [45]. Moreover, miR-145 has been reported to target the lncRNA taurine upregulated 1 (TUG1), a tumor oncogene associated with various human cancers. The deregulated ceRNA network of the TUG1/miR-145/zinc finger E-box binding homeobox 1 (ZEB1) signaling pathway increased cell proliferation, enhanced migration capacity, and promoted EMT formation in three thyroid cancer cell lines (the ATC cell lines SW1736 and KAT18 and the FTC cell line FTC133) [46]. In contrast, metastasis and proliferation were suppressed in cells transfected with miR-145 mimics. Overexpression of miR-145 may reverse the EMT phenotype of thyroid cancer cells; relative levels of miR-145 in thyroid cancer tissues were significantly lower than in the corresponding normal tissues [46]. Moreover, Wang et al., testing genetic variants flanking the miR-145 gene, found significant association between rs4705342*T single nucleotide polymorphism and a higher risk of papillary thyroid carcinoma [48].

### 3.5. Functional Role of miR-145 in Cancer

**Figure 8:**
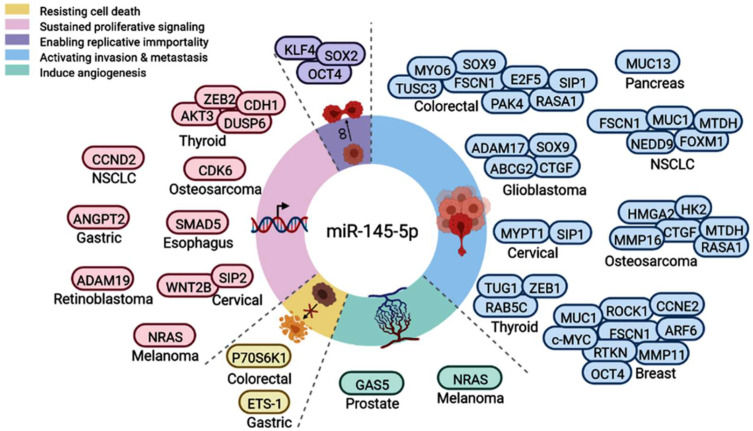
Functional role of miR-145-5p in hallmarks of cancer. Upregulated genes/proteins targeted by miR-145-5p in various types of cancers are shown, leading to resistance to cell death, sustained proliferative signaling, activating invasion and metastasis, or inducing angiogenesis. Pie chart represents the proportion of articles demonstrating these cancer hallmarks.

Functional enrichment analysis of miR-145 showed connections with multiple cancer-related mechanisms and pathways. Specifically, miR-145-5p can negatively regulate multiple genes in the thyroid cancer signaling pathway (hsa05216, *p* = 0.031), including tropomyosin 3 (TPM3), neuroblastoma RAS proto-oncogenes (NRAS), catenin beta 1 (CTNNB1), MYC proto-oncogene, BHLH transcription factor (MYC), and cyclin D1 (CCND1). A systematic review of the literature revealed miR-145′s role in various hallmarks of cancer (Figure 8); for example, it suppresses cell invasion and metastasis in breast cancer cell lines by directly targeting mucin 1 (MUC1) [50]. Moreover, miR-145-5p negatively regulated SMAD5 in esophageal cancer, which in turn can activate the TGF beta signaling pathway. High expression of miR-145 decreased angiogenesis [51]; induced differentiation of macrophages by interleukin 6 (IL-6) [52]; silenced the stem cell self-renewal and pluripotency program by suppressing multiple pluripotent genes such as OCT4, SOX2, and KLF4 [53]; promoted the epithelial–mesenchymal transition by suppressing FGF10 [54]; and increased apoptosis in prostate cancer [55].

The transcription factors FLI1, GATA1, PPARG, RAD21, RCOR3, SIN3B, SPI1, SREBF2, STAT3, STAT5A, and TP63 were found to target the promoter of the MIR145 gene. We did not find any of these markers to be deregulated in the thyroid cancer TCGA dataset. Using ingenuity pathway analysis, gene ontology analysis of the MIR145 gene showed subcellular localization in extracellular vesicular exosomes and RNA-induced silencing complex in cytoplasm. Some biological processes included actin cytoskeleton organization, cell differentiation, negative regulation of angiogenesis, negative regulation of cell migration, negative regulation of inflammatory response, positive regulation of canonical Wnt receptor signaling pathway, positive regulation of cardiac vascular smooth muscle cell differentiation, positive regulation of fibroblast migration, positive regulation of macrophage activation, positive regulation of macrophage differentiation, regulation of collagen biosynthetic process, regulation of ERK1 and ERK2 cascade, transforming growth factor beta receptor signaling pathway, and vascular smooth muscle cell differentiation.

## 4. Discussion

**Figure 9:**
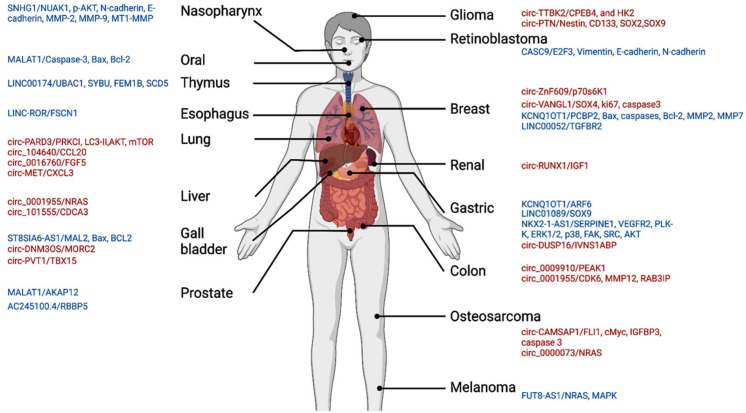
MiR-145 is sponged by circRNA (red) and lncRNAs (blue) in various types of cancer. Slashes separate circRNA or lncRNA from downstream targets.

The MIR145 gene is located at the 5p32 chromosomal region (5:149, 430, 646–149, 430, 733 forward strand). Its expression is regulated by p53 and other transcriptional factors [56]. The tumor-suppressive role of miR-145 has been suggested in diverse types of cancers [57,58,59], such as breast cancer [60], gastric cancer [61], bladder cancer [62], colorectal cancer [63,64], and ovarian cancer [65]. It has been sponged by multiple circular RNAs and lncRNAs, leading to deregulation of downstream gene targets (Figure 9). Previous studies have indicated that miR-145 is associated with cell apoptosis, proliferation, stem cell differentiation, angiogenesis, and metastasis [50,51,52,54,55].

In thyroid cancer, research has indicated that miR-145 is a downregulated tumor suppressor miRNA. Functional and preclinical studies identified AKT3, ZEB1, ZEB2, DUSP6, and RAB5C as direct targets of miR-145 [41,43,44,45,46]. Akt is a crucial protein of the PI3K/Akt signal transduction cascade that plays a central role in cell proliferation, apoptosis, and motility in thyroid cancer [66]. ZEB1 functions to regulate important transcriptional networks necessary for cell differentiation, maintenance, and function [67]. It can modulate the PD-1/PD-L1 checkpoint [68,69], promote cell proliferation and migration, and inhibit apoptosis [70]. It also plays a crucial role in the maintenance of cancer stem cells [71]. ZEB2 functions as a DNA-binding transcriptional repressor that interacts with activated SMADs [72]. ZEB2 can trigger the induction of genes associated with epithelial–mesenchymal transition [73], activate the Wnt/beta-catenin pathway [74], and regulate invasion and metastasis [72]. Endosomal RAB5C was reported to prevent VEGFR2 degradation during angiogenesis [75], regulate focal adhesion-mediated cell migration [76], and enhance resistance to ionizing radiation [77]. DUSP6, a member of the MAPK phosphatase family, is highly expressed in PTC and plays a pro-oncogenic role in thyroid tumorigenesis by regulating the ERK1/2 pathway, cell proliferation, and invasiveness [78]. Studies investigating the mechanism responsible for the aberrant expression of miR-145 in thyroid cancer demonstrated the role of three ncRNAs (TUG1, n384546, and circNUP214) in suppressing miR-145 expression [45,46,47]. Additionally, miR-145 was found to modulate NF-κB pathway regulation in PTC cells [49].

Recent studies demonstrated that molecular profiling of miR-145 is emerging as a key noninvasive tool for monitoring cancer invasion and migration [79]. Interestingly, miR-145 has been detected in peripheral blood and other body fluids [80]. Expression levels of miR-145–5p have been correlated with clinical parameters in cancer patients, as shown in Table 4. In the present study, the expression of miR-145 was analyzed in 284 paired tissues and 111 blood samples. We found that miR-145-5p was downregulated in cancer tissues and blood compared with counterparts. Additionally, a lower miR-145 level was associated with advanced clinical stage, larger tumor size, and lymph node metastasis. Similarly, in the TCGA data, low tissue expression of miR-145-5p was associated with a higher tumor stage, distant metastasis, extrathyroidal extension, and BRAF^V600E^ mutation.

Several chemical compounds used as medications have been shown to influence the expression pattern of miR-145-5p. Propofol modulates the proliferation, invasion, and migration of bladder cancer cells through the miR-145-5p/TOP2A axis [95]. In glioma, ropivacaine could promote cell apoptosis and inhibit tumor growth, proliferation, migration, and invasion through the regulation of the circ-SCAF11/miR-145-5p axis [96]. Apoptosis-related miR-145-5p enhanced the effects of pheophorbide a-based photodynamic therapy (Pa-PDT) in oral cancer [97]. Bupivacaine via targeting circ_0000376 and miR-145-5p up-regulation could cause cell apoptosis in gastric cancer cells [98]. Therefore, assessment of expression of this miRNA is a potential way for prediction of the response to these medications in different conditions.

As a tumor suppressor miRNA, miR-145 replacement therapy caused profound antitumor effects in colon carcinoma and metastatic castration-resistant prostate cancer animal models [99,100]. Similarly, miR-145-5p was reported to enhance the therapeutic response to some chemotherapeutic agents such as sorafenib in HCC [101], methotrexate in osteosarcoma [102], and docetaxel in prostate cancer [103]. Moreover, cumulative evidence showed that miR-145 acts to reverse therapeutic resistance in various tumors [104]; Table 5. For example, MiR-145 changes the sensitivity of non-small cell lung cancer to gefitinib through targeting ADAM19 [105]. miR-145 sensitizes esophageal squamous cell carcinoma to cisplatin through directly inhibiting the PI3K/AKT signaling pathway [106]. miR-145 antagonizes SNAI1-mediated stemness and radiation resistance in colorectal cancer [107], and reversed 5-fluorouracil (5-FU) drug resistance by directly targeting DNA damage-related gene RAD18 in colorectal cancer [108]. MicroRNA 145 enhances the chemosensitivity of glioblastoma stem cells to desmethoxycurcumin [109], thus suggesting that it might serve as a candidate and promising biomarker for drug resistance and that therapeutic up-regulation of this miRNA might be suggested as a modality for enhancing the response to conventional as well as targeted therapies.

## 5. Conclusions

This work is the first to indicate putative regulatory networks and mechanisms regulating its suppressive role in tumorigenesis and progression. Taken together, these findings suggest that miR-145 is a key regulator of thyroid cancer growth, mediates its effect through multiple gene targets and pathways, is secreted by the thyroid cancer cells, and may serve as an adjunct biomarker for TC diagnosis and prognosis. Genome-wide screening of miR-145 gene targets in in vitro and in vivo studies will be necessary for further exploration of the potential role of miR-145 in TC progression. Further large-scale, multicenter studies are needed to better elucidate the dynamic changes in miR-145 during surveillance follow-up; however, our comprehensive analysis provides evidence that miR-145 expression is downregulated in TC tissues and blood, and its expression pattern is significantly negatively associated with poor prognosis.

## Figures and Tables

**Table 1:** Characteristics of the study population (*n* = 467).

Characteristics	Levels	Total	Disease-Free	Recurred/Progressed	*p*-Value
Demographic data					
Age, years	Median (IQR)	46.0 (34.0–57.0)	46.0 (34.0–56.0)	51.5 (32.7–63.0)	0.21
	<55 y	321 (68.7)	297 (70.2)	24 (54.5)	**0.040**
	≥55 y	146 (31.3)	126 (29.8)	20 (45.5)	
Sex	Female	342 (73.2)	313 (74)	29 (65.9)	0.28
	Male	125 (26.8)	110 (26)	15 (34.1)	
Ethnicity	Not Hispanic or Latino	328 (70.2)	292 (69)	36 (81.8)	0.17
	Hispanic or Latino	36 (7.7)	33 (7.8)	3 (6.8)	
	Missing	103 (22.1)	98 (23.2)	5 (11.4)	
Race	White	312 (66.8)	279 (66)	33 (75)	**0.018**
	Asian	48 (10.3)	44 (10.4)	4 (9.1)	
	Black	16 (3.4)	15 (3.5)	1 (2.3)	
	American Indian or Alaska Native	1 (0.2)	0 (0)	1 (2.3)	
	Missing	90 (19.3)	85 (20.1)	5 (11.4)	
Pathological assessment					
Primary tumor laterality	Right lobe	201 (43)	183 (43.3)	18 (40.9)	0.87
	Left lobe	161 (34.5)	145 (34.3)	16 (36.4)	
	Bilateral	81 (17.3)	72 (17)	9 (20.5)	
	Isthmus	20 (4.3)	19 (4.5)	1 (2.3)	
	Missing	4 (0.9)	4 (0.9)	0 (0)	
Focality	Unifocal	247 (52.9)	223 (52.7)	24 (54.5)	0.72
	Multifocal	214 (45.8)	194 (45.9)	20 (45.5)	
	Missing	6 (1.3)	6 (1.4)	0 (0)	
Histopathology type	Papillary	363 (77.7)	325 (76.8)	38 (86.4)	0.18
	Follicular	104 (22.3)	98 (23.2)	6 (13.6)	
Pathology Stage	Stage I	271 (58)	252 (59.6)	19 (43.2)	0.051
	Stage II	48 (10.3)	45 (10.6)	3 (6.8)	
	Stage III	99 (21.2)	86 (20.3)	13 (29.5)	
	Stage IV	47 (10.1)	38 (9)	9 (20.5)	
	Missing	2 (0.4)	2 (0.5)	0 (0)	
T stage	T1a	18 (3.9)	17 (4)	1 (2.3)	**0.009**
	T1b	120 (25.7)	117 (27.7)	3 (6.8)	
	T2	155 (33.2)	141 (33.3)	14 (31.8)	
	T3	155 (33.2)	133 (31.4)	22 (50)	
	T4a	17 (3.6)	13 (3.1)	4 (9.1)	
	Missing	2 (0.4)	2 (0.5)	0 (0)	
N stage	N0	215 (46)	201 (47.5)	14 (31.8)	0.053
	N1a	86 (18.4)	78 (18.4)	8 (18.2)	
	N1b	123 (26.3)	104 (24.6)	19 (43.2)	
	Nx	43 (9.2)	40 (9.5)	3 (6.8)	
M stage	M0	257 (55)	237 (56)	20 (45.5)	**<0.001**
	M1	8 (1.7)	4 (0.9)	4 (9.1)	
	Mx	202 (43.3)	182 (43)	20 (45.5)	
Extrathyroidal extension	Negative	318 (68.1)	295 (69.7)	23 (52.3)	0.06
	Minimal	122 (26.1)	103 (24.3)	19 (43.2)	
	Advanced	13 (2.8)	12 (2.8)	1 (2.3)	
	Missing	14 (3)	13 (3.1)	1 (2.3)	
Oncologic assessment					
BRAF mutation	Wild type	87 (18.6)	80 (18.9)	7 (15.9)	0.17
	Mutant	225 (48.2)	198 (46.8)	27 (61.4)	
	Missing	155 (33.2)	145 (34.3)	10 (22.7)	
TERT mutation	Wild type	329 (70.4)	305 (72.1)	24 (54.5)	**0.011**
	Mutant	31 (6.6)	24 (5.7)	7 (15.9)	
	Missing	107 (22.9)	94 (22.2)	13 (29.5)	
Mutation density	Median (IQR)	0.51 (0.31–51.9)	0.51 (0.31–0.70)	0.64 (0.35–0.88)	0.07
ATA risk group	Low	127 (27.2)	121 (28.6)	6 (13.6)	**0.002**
	Intermediate	233 (49.9)	214 (50.6)	19 (43.2)	
	High	107 (22.9)	88 (20.8)	19 (43.2)	
Intervention					
Radioactive iodine	Negative	191 (40.9)	171 (40.4)	20 (45.5)	0.36
	Positive	17 (3.6)	17 (4)	0 (0)	
Radiation treatment	Negative	68 (14.6)	65 (15.4)	3 (6.8)	0.20
	Positive	140 (30)	123 (29.1)	17 (38.6)	
Follow-up					
Mortality	Survived	465 (99.6)	423 (100)	42 (95.5)	**0.009**
	Died	2 (0.4)	0 (0)	2 (4.5)	
Overall survival, months	Median (IQR)	31.0 (17.4–51.9)	30.9 (16.8–50.2)	42.1 (22.9–71.9)	**0.006**

Data are represented as frequency (percentage), mean ± standard deviation (SD), or median and interquartile range (IQR). BMI: body mass index, LN: lymph node, EBRT: external beam radiation therapy. Two-sided Chi-square test, Student’s *t*-test, and Mann–Whitney U test were used. Bold values indicate significance at *p*-value < 0.05.

**Table 2:** Expression levels of miR-145-5p in liquid biopsy samples.

**Cancer Type**	**Source ID**	**Log Fold Change**	**Expression Status**	**Design**
Biliary tract cancer	GSE59856	−0.21	DOWN	blood
Brain cancer	SRP262521	−2.09	DOWN	blood
	GSE113740	1.74	UP	blood
	GSE113486	2.06	UP	blood
	GSE112264	2.52	UP	blood
	GSE139031	2.82	UP	blood
Breast cancer	GSE113486	1.43	UP	blood
	GSE106817	1.51	UP	blood
Colon cancer	GSE39845	1.5	UP	blood
Colorectal cancer	GSE106817	1.96	UP	blood
Esophageal cancer	GSE59856	−0.36	DOWN	blood
	GSE112840	−0.04	DOWN	blood
	GSE113486	1.23	UP	blood
	GSE122497	1.82	UP	blood
	GSE106817	2.3	UP	blood
Gastric cancer	GSE113740	1.5	UP	blood
	GSE106817	1.55	UP	blood
	GSE113486	1.72	UP	blood
Head and neck cancer	SRP078325	1.6	UP	exosomes
Hepatocellular carcinoma	GSE113740	0.97	UP	blood
	GSE106817	1.34	UP	blood
Leukemia	E_MTAB_1454	−0.64	DOWN	blood
Liver cancer	GSE59856	−0.48	DOWN	blood
Lung cancer	GSE113486	1.47	UP	blood
	GSE112264	1.59	UP	blood
	GSE106817	2.42	UP	blood
Lymphoma	GSE139031	1.85	UP	blood
Melanoma	SRP262521	−0.84	DOWN	blood
	GSE31568	1.6	UP	blood
Ovarian cancer	GSE113740	1.6	UP	blood
	GSE113486	1.96	UP	blood
	GSE106817	1.73	UP	blood
Pancreatic cancer	GSE113486	1.56	UP	blood
	GSE113740	1.66	UP	blood
	GSE112264	1.71	UP	blood
	GSE106817	1.89	UP	blood
Prostate cancer	GSE31568	1.26	UP	blood
Sarcoma	GSE65071	−1.22	DOWN	blood
	GSE106817	1.1	UP	blood

**Table 3:** Baseline characteristics of the study population.

Characteristics	Levels	FFPE Samples (*n* = 178)		*p*-Value	Frozen and Blood Samples (*n* = 64)		*p*-Value
		Non-Recurrence (*n* = 138)	Recurrence (*n* = 40)		Non-Recurrence (*n* = 56)	Recurrence (*n* = 8)	
Demographic data							
Age, years	Median (IQR)	45 (33.7–56.2)	51.5 (33.5–62.7)	0.19	41 (34–52)	49.5 (32.0–60.7)	0.59
	<55 y	96 (69.6)	21 (52.5)	0.06	43 (76.8)	4 (50)	0.19
	≥55 y	42 (30.4)	19 (47.5)		13 (23.2)	4 (50)	
Sex	Female	95 (68.8)	25 (62.5)	0.45	48 (85.7)	6 (75)	0.60
	Male	43 (31.2)	15 (37.5)		8 (14.3)	2 (25)	
Ethnicity	Not Hispanic or Latino	138 (100)	40 (100)	NA	41 (73.2)	7 (87.5)	0.66
	Hispanic or Latino	--	--		15 (26.8)	1 (12.5)	
Race	White	138 (100)	40 (100)	NA	12 (21.4)	2 (25)	0.66
	Black	--	--		14 (25)	3 (37.5)	
	Asian	--	--		30 (53.6)	3 (37.5)	
Hashimoto disease	Positive	42 (30.4)	13 (32.5)	0.84	15 (26.8)	1 (12.5)	0.67
Pathological assessment							
Focality	Unifocal	82 (59.4)	22 (55)	0.71	35 (62.5)	4 (50)	0.70
	Multifocal	56 (40.6)	18 (45)		21 (37.5)	4 (50)	
Histopathology type	Follicular	24 (17.4)	5 (12.5)	0.62	8 (14.3)	2 (25)	0.60
	Papillary	114 (82.6)	35 (87.5)		48 (85.7)	6 (75)	
Pathology stage	Stage I	80 (58)	14 (35)	**0.001**	40 (71.4)	4 (50)	0.08
	Stage II	19 (13.8)	2 (5)		3 (5.4)	0 (0)	
	Stage III	32 (23.2)	16 (40)		9 (16.1)	1 (12.5)	
	Stage IV	7 (5.1)	8 (20)		4 (7.1)	3 (37.5)	
T stage	T1	39 (28.3)	2 (5)	**<0.001**	14 (25)	1 (12.5)	0.40
	T2	55 (39.9)	12 (30)		18 (32.1)	1 (12.5)	
	T3	42 (30.4)	23 (57.5)		20 (35.7)	5 (62.5)	
	T4	2 (1.4)	3 (7.5)		4 (7.1)	1 (12.5)	
N stage	N0	82 (59.4)	14 (35)	**0.007**	25 (44.6)	4 (50)	0.77
	N1	56 (40.6)	26 (65)		31 (55.4)	4 (50)	
M stage	M0	132 (95.7)	35 (87.5)	0.07	55 (98.2)	6 (75)	**0.039**
	M1	6 (4.3)	5 (12.5)		1 (1.8)	2 (25)	
Extrathyroidal extension	Negative	100 (72.5)	19 (47.5)	**0.004**	32 (57.1)	5 (62.5)	0.77
	Positive	38 (27.5)	21 (52.5)		24 (42.9)	3 (37.5)	
Oncologic assessment							
*BRAF* mutation	Wild type	73 (52.9)	16 (40)	0.15	20 (35.7)	3 (37.5)	0.92
	Mutant	65 (47.1)	24 (60)		36 (64.3)	5 (62.5)	
*TERT* mutation	Wild type	66 (47.8)	0 (0)	**<0.001**	48 (85.7)	7 (87.5)	0.89
	Mutant	70 (50.7)	34 (85)		8 (14.3)	1 (12.5)	
Intervention							
Radioactive iodine	Positive	1 (0.7)	0 (0)	0.59	3 (5.4)	1 (12.5)	0.42
Radiation treatment	Positive	31 (22.5)	13 (32.5)	0.21	18 (32.1)	1 (12.5)	0.25
Follow-up							
Mortality	Survived	138 (100)	39 (97.5)	0.23	55 (98.2)	8 (100)	0.70
	Died	0 (0)	1 (2.5)		1 (1.8)	0 (0)	
Overall survival, months	Median (IQR)	37.9 (21.6–63.3)	35.6 (24.0–63.7)	0.88	31.8 (14.3–58.4)	43.1 (32.8–50.0)	0.22
Disease-free survival, months	Median (IQR)	37.9 (21.6–63.4)	13.4 (7.1–25.2)	**<0.001**	31.8 (14.2–58.1)	21.4 (7.0–43.6)	0.33

Data are represented as frequency (percentage) or median and interquartile range (IQR). Two-sided chi-square and Mann–Whitney U tests were used. Bold values indicate significance at *p*-value < 0.05.

**Table 4:** Association between miRNA and clinical parameters in cancer patients.

Cancer Type	Sample	Size (Cancer/Control)	Grade	T Stage	N Stage	TNM	Survival	Reference
Bladder	Tissues	22/22			●			[81]
Breast	Blood	35/33	●					[82]
Cervical	Tissue	40/40	●		●			[83]
Esophagus	Tissue	30/30					●	[84]
Gall bladder	Tissue	40/8					●	[85]
Gastric	Tissue	289/0					●	[86]
Gastric	Tissue	60/60			●	●		[87]
Glioblastoma	Blood	117/0					●	[88]
Liver	Tissues	60/60					●	[89]
Liver	Tissues	150/150	●	●	●	●	●	[90]
Liver	Tissues	10/10					●	[91]
Larynx	Tissue	188/0	●	●	●	●	●	[92]
Melanoma	Tissue	83/83		●		●		[93]
Ovarian	Tissue	414/0				●	●	[93]
Prostate	Tissue	64/64				●		[94]
Prostate	Blood	64/55				●		[94]

Dot indicate positive association.

**Table 5:** The role of miR-145 enhances cancer therapy sensitivity and reverse resistance.

Therapy	Cancer Type	Downstream Targets	Reference
5-FU	Colorectal cancer	RAD18	[108]
	Esophageal carcinoma	REV3L	[110]
Bortezomib	Multiple myeloma	HDAC4	[111]
Cisplatin	Gastric cancer	APRIL	[112]
	Ovarian cancer	c-Myc	[113]
	Gallbladder cancer	MRP1	[114,115]
	Nasopharyngeal carcinoma	SOX2	[116]
	Non-small cell lung cancer	MRP1 and P-gp	[117]
		CDK6	[118]
		KLF4	[119]
	Esophageal carcinoma	MRP1 and P-gp	[106]
Docetaxel	Lung adenocarcinoma	FSCN1	[120]
	Prostate cancer	AKAP12	[103]
Doxorubicin	Hepatocellular carcinoma	SMAD3	[121]
	Breast cancer	MRP1	[122]
Erlotinib	Non-small cell lung cancer	EGFR	[123]
Gefitinib	Non-small cell lung cancer	ADAM19	[105]
Gemcitabine	Bladder cancer	HMGA2 and KLF4	[124]
	Pancreatic adenocarcinoma	P70S6K1	[125]
Imatinib	Hepatocellular carcinoma	P-gp and BCRP	[126]
Oxaliplatin	Colorectal cancer	GPR98	[127]
		MRP1	[128]
Paclitaxel	Ovarian cancer	Sp1 and CDK6	[65]
Radiation	Cervical cancer	HLTF	[129]
	Colorectal cancer	KLF4 and c-Myc	[107]
	Esophageal carcinoma	P70S6K1	[130]
	Prostate cancer	RAD51, Mcl1, Par-4, and PARP1	[127]
	Hepatocellular carcinoma	RAD18	[131]

## Data Availability

Data are available from the corresponding author upon reasonable request and obtaining the approval of the “Office of Technology Transfer and Intellectual Property Development, Tulane University, USA”. Sources of in silico data analysis sections are available in public repositories through the links provided in the manuscript.
